# Can Social Protection Improve Sustainable Development Goals for Adolescent Health?

**DOI:** 10.1371/journal.pone.0164808

**Published:** 2016-10-17

**Authors:** Lucie D. Cluver, F. Mark Orkin, Franziska Meinck, Mark E. Boyes, Alexa R. Yakubovich, Lorraine Sherr

**Affiliations:** 1 Centre for Evidence-Based Intervention, Department of Social Policy & Social Intervention, University of Oxford, Oxford, United Kingdom; 2 Department of Psychiatry and Mental Health, University of Cape Town, Cape Town, South Africa; 3 DPHRU, School of Clinical Medicine, and DST-NRF Centre of Excellence in Human Development, University of the Witwatersrand, Johannesburg, South Africa; 4 Health Psychology and Behavioural Medicine Research Group, School of Psychology and Speech Pathology, Curtin University, Perth, Australia; 5 Department of Infection & Population Health, University College London, London, United Kingdom; Institute for Health & the Environment, UNITED STATES

## Abstract

**Background:**

The first policy action outlined in the Sustainable Development Goals (SDGs) is the implementation of national social protection systems. This study assesses whether social protection provision can impact 17 indicators of five key health-related SDG goals amongst adolescents in South Africa.

**Methods:**

We conducted a longitudinal survey of adolescents (10–18 years) between 2009 and 2012. Census areas were randomly selected in two urban and two rural health districts in two South African provinces, including all homes with a resident adolescent. Household receipt of social protection in the form of ‘cash’ (economic provision) and ‘care’ (psychosocial support) social protection, and health-related indicators within five SDG goals were assessed. Gender-disaggregated analyses included multivariate logistic regression, testing for interactions between social protection and socio-demographic covariates, and marginal effects models.

**Findings:**

Social protection was associated with significant adolescent risk reductions in 12 of 17 gender-disaggregated SDG indicators, spanning SDG 2 (hunger); SDG 3 (AIDS, tuberculosis, mental health and substance abuse); SDG 4 (educational access); SDG 5 (sexual exploitation, sexual and reproductive health); and SDG 16 (violence perpetration). For six of 17 indicators, combined cash plus care showed enhanced risk reduction effects. Two interactions showed that effects of care varied by poverty level for boys’ hunger and girls’ school dropout. For tuberculosis, and for boys’ sexual exploitation and girls’ mental health and violence perpetration, no effects were found and more targeted or creative means will be needed to reach adolescents on these challenging burdens.

**Interpretation:**

National social protection systems are not a panacea, but findings suggest that they have multiple and synergistic positive associations with adolescent health outcomes. Such systems may help us rise to the challenges of health and sustainable development.

## Introduction

Last year, the Millennium Development Goals expired. Replacing them are the Sustainable Development Goals (SDGs): an ambitious 17 overarching goals with targets and indicators that UN member states will use to guide human development policy over the next fifteen years. If the SDGs are to move from aspirations to an implementable strategy, evidence-based interventions need to be rapidly decided upon and scaled up [1]. The SDGs identify poverty and inequality as major barriers to health for the world’s most structurally deprived populations. They combine ambitious human development outcomes, such as eliminating hunger and gender disparities, with ‘policy actions’ designed to achieve those outcomes.

In doing so, the SDGs offer both great challenges and potential solutions. The first identified policy action, SDG 1.3, is to ‘implement nationally appropriate social protection systems and measures for all and by 2030 achieve substantial coverage of the poor and vulnerable’. Definitions of social protection vary, but often follow Devereux and Sabates-Wheeler: ‘Social protection describes all public and private initiatives that provide income or consumption transfers to the poor, protect the vulnerable against livelihood risks, and enhance the social status and rights of the marginalised; with the overall objective of reducing the economic and social vulnerability of poor, vulnerable and marginalised groups’. Importantly, they clarify that social protection can be provided in both formal ‘public and private’, and by informal ‘collective or community-level’ sources [2].

A key vulnerable group are adolescents in Africa. Despite many advances, Sub-Saharan Africa remains the region with the lowest Human Development Index, lowest life expectancy, and greatest health and gender inequalities.[3] In the past decade, South Africa has emerged as the world’s least economically equal society,[4] with persisting racial divides.[3] Adolescents and youth are hard-hit: HIV-infection rates amongst young African females are rising,[5] as are violent deaths amongst young males,[6] and sexual violence remains amongst the highest in the world.[7]

For these adolescents, social protection may offer both scaleability and potential. Existing research focuses on government-provided cash transfers, which have been shown to improve mental health, education and sexual health [8], and whilst debate remains around conditionality of such transfers, most governments in the region have favoured unconditional or ‘soft’ conditioned provision. However, cash transfers may not be enough. For example in South Africa, cash showed protective effects against HIV-risk behaviors for girls, but less or no impacts on boys [9, 10]. Subsequent HIV-focused studies then tested whether the addition of other types of social protection provisions may increase the impact of cash transfers. In South Africa, cash and other types of economic support such as school feeding were tested in combination with psychosocial care provisions such as positive parenting, good supervision from a primary caregiver or teacher support. The combination of ‘cash plus care’ further protected girls from HIV-risk behaviors and additionally reached boys. [11] For education outcomes, cash transfers have also been shown to benefit girls more than boys, but cash and care combinations have not been investigated [12].

Cash transfers and care programmes are being implemented in many low-income settings, particularly in Africa and South America, but many programmes remain small-scale. ‘Cash’ and ‘care’ can take many forms, are focused at different beneficiary groups, and differ in extent between and within countries–for example Kenya’s main cash transfer is targeted at households of orphaned and vulnerable children, whilst South Africa’s is for all low-income families [10]. Countries such as Malawi, Tanzania and Uganda provide free primary schooling, but concerns remain about continuing fees, costly textbooks and lack of secondary education provision [13]. Psychosocial care for children and adolescents is often received in the home or school setting, following evidence that family-based support is the most sustainable and effective approach for child development [14], but this often requires external support for struggling or vulnerable caregivers–primarily from NGOs and including a range from general social support to structured parenting programmes [15].

State adoption of broader social protection policies would thus represent a considerable political and fiscal undertaking.[16]. A cost-benefit analysis of a cash transfer delivered as part of a randomised trial in Malawi showed HIV, mental health and education benefits for adolescent girls. If governments considered co-financing between departmental budgets, benefits outweigh costs for each sector [17]. With the advent of the SDGs, comprising 17 new goals with a wide range of targets and suggested indicators, it is opportune to examine whether cash alone, care alone or cash plus care has traction with more than one of the health-relevant SDG outcomes. This would help identify pathways for structural intervention whereby multiple benefits could be anticipated. It is also important to understand whether social protection shows different patterns of effect for boys and girls: Gender equality is itself an SDG 5, but a step in achieving this requires gender-disaggregated examination of potential programming.

This South African study presents an opportunity to test these potential impacts.[18] Between 2009–2012, the government extended the receipt of child-focused cash transfers to adolescents from an age-limit of 12 to 18. Additionally, roll-out of free schooling and school meals was underway for highest-deprivation districts. Simultaneously, international organisations were expanding provision of psychosocial ‘care’ support to adolescents, through NGO and parenting support. During this period, access and take-up of all of these interventions was uneven, as typifies large-scale programming and family-based care in any resource-constrained context. This uneven scale-up of both ‘cash’ and ‘care’ social protection allowed rigorous testing of associations with health outcomes in a real-world African context. Given that ‘natural experiments’ are quasi-experimental rather than randomised designs, it is also desirable to check whether social protection effects are modified by interactions with each other or with any socio-demographic factors that also predict adolescent health outcomes.

This study thus has three aims: 1) to test associations of social protection and indicators for health-relevant targets of five SDG goals, amongst highly deprived African adolescent boys and girls; 2) to test for potential interactions between social protection and socio-demographic co-predictors of adolescent health; 3) to test where ‘cash’ and/or ‘care’ forms of social protection are effective, or where combined ‘cash plus care’ can provide additive benefits.

## Methods

### Participants and procedures

3515 adolescents aged 10–18 (56.7% female) were interviewed at baseline (2009–10) and followed up at one year (2011–12). Baseline refusal rate was <2.5% and retention rate 96.8%. Two urban and two rural health districts, all low-income, high HIV-prevalence and majority black African, were selected within two South African provinces: Mpumalanga and the Western Cape. Within each health district, census enumeration areas were randomly sampled until sample size was reached. In each area, every household was visited and included in the study if they had a resident adolescent. One randomly-selected adolescent per household was interviewed face-to-face for 60–70 minutes. Questionnaires and consent forms were translated and checked with back-translation into Xhosa, Zulu, Sotho, Swati and Tsonga, and adolescents chose their language of participation.

Ethical protocols were approved by university Institutional Review Boards of Oxford, Cape Town and KwaZulu-Natal, and by provincial Health, Education and Social Development Departments. Voluntary written informed consent was obtained from adolescents and primary caregivers, and to ensure full understanding of the study, information and consent processes were additionally read aloud to all participants. No incentives were given apart from refreshments and certificates of participation. Interviewers were trained in working with vulnerable youth and confidentiality was maintained except when participants were at risk of significant harm or requested assistance. Where adolescents reported recent abuse, rape, or risk of significant harm, referrals were made to child protection, HIV/AIDS, and health services, with follow-up support.

### Outcome measures

Five SDGs were identified as highly relevant to adolescent health: hunger (SDG 2), health (SDG 3), education (SDG 4), gender equality (SDG 5), and peaceful societies (SDG 16). Within each goal, indicators of negative health outcomes were selected that were measurable and had potential to be impacted by social protection. All measures were taken at baseline and follow-up.

#### SDG 2 ‘End hunger, achieve food security’

*Adolescent hunger* (SDG 2.1) was measured using SA National Food Consumption Survey items[19] and defined as experiencing more than one day of insufficient food in the home during the past week.

#### SDG 3 ‘Ensure healthy lives’

*HIV-risk behaviour* (SDG 3.3) was assessed as one or more of four high-risk behaviours from the National Survey of HIV and Sexual Behaviour amongst Young South Africans.[20, 21] Unprotected sex was inconsistent/no condom use during past year sex; multiple sexual partners was three or more past-year partners,[22] sex whilst using substances included inebriation or any drug use; early debut was initiation of sex below age 15. *Tuberculosis* (SDG 3.3) was measured as pulmonary tuberculosis disease using an 8-item WHO symptom checklist.[23] A conservative threshold was set of four or more symptoms of fever, discoloured sputum, fatigue, weight loss, and night sweats in addition to two or more symptoms of coughing blood, chest pains, and cough for more than three weeks.[24] *Mental health risk* (SDG 3.4) used standardized psychometric tools, measuring one or more clinical-level disorder of depression (Child Depression Inventory Short Form),[25] anxiety (Revised Children’s Manifest Anxiety Scale),[26] or suicidality (MINI International Psychiatric Interview for Children and Adolescents).[27] *Substance and alcohol misuse* (SDG 3.5) used 15 items from the National Survey of HIV and Risk Behaviour [21] and included past-year weekly or more frequent alcohol use, inebriation, and any drug use including marijuana, mandrax or crystal methamphetamine.

#### SDG 4 ‘Inclusive and equitable education’

*School non-enrolment* (SDG 4.1) was school dropout (prior to completion of senior school) due to any cause.

#### SDG 5 ‘Achieve gender equality and empower women and girls’

*Sexual violence and exploitation of girls* (SDG 5.2) was any of past-year sexual abuse, rape (using UNICEF child protection scales),[28] transactional sexual exploitation (sex in exchange for food, shelter, school fees, transport, or money) or age-disparate sex (sexual partner more than five years older than the adolescent).[21] *Lack of access to sexual and reproductive health* (SDG 5.6) was measured for adolescent girls only, as pregnancy or childbirth prior to age 17.

#### SDG 16: ‘Promote peaceful and inclusive societies’

*Adolescent violence perpetration* (SDG 16.1) was measured using violence items from the delinquency and aggression subscales of the Child Behaviour Checklist,[29, 30] and included past-month robbery, vandalism, carrying of a knife or a gun.

#### SDG 1.3: Social Protection

Due to strong evidence that both cash provision and psychosocial care require sustained and predictable duration in order to maintain effects on child development,[31] each type of cash and care was coded positively only if received at both baseline and one-year follow-up. Variables measured receipt of ‘cash only’, ‘care only’, and ‘cash plus care’.

‘Cash’ was grouped as either direct cash transfers or ‘in kind’ transfers of free education and food, following evidence that families use cash primarily for food and school expenses.[32] Thus, ‘cash’ social protection was measured as accessing one or more of child-focused cash transfer (household access to either a government Child Support or Foster Child grant),[33] or free schooling (free school and textbooks) and school feeding (daily, free school-provided meals).

Access to ‘care’ social protection was sustained receipt of ≥1 of positive parenting (e.g. primary caregiver praise and warmth) and good parental monitoring (e.g. household rules and consistent supervision), measured using the Alabama Parenting Questionnaire,[34] and teacher social support (social, practical and emotional) using a standardized scale [35] and dichotomized as ‘high support’.

### Covariates

Covariate**s** included both baseline socio-demographics and potential confounders of social protection access or SDG outcomes, including predictors for grant receipt. These included adolescent age (>13), urban/rural site (using census definitions), and informal housing. Poverty was measured using the SA Social Attitudes survey’s basic necessities for children and coded as missing more than two necessities [36]. Household employment whether anyone in the household had a job (full or part-time). Number of children (>2) in the household used a ‘household map’. Female primary caregiver was measured, with primary caregiver identified as ‘the person who lives with you and looks after you most’. Possession of a birth certificate was measured as a potential predictor of cash and school access.

### Analyses

Analyses were conducted in five stages disaggregated by gender using SPSS 22 and Stata 13. First, we tested for differences between youth lost and retained at follow-up on baseline socio-demographic characteristics, SDG health indicators, and social protection access. Second, using the sample of adolescents retained across both time points (n = 3401, 97%), we examined socio-demographics, negative SDG health outcomes, and social protection access. Third, we tested associations between each SDG indicator and dummy variables for ‘cash’ and ‘care’ social protection, following the sequential approach recommended by Hosmer and Lemeshow [37]. For each SDG indicator at follow-up as the outcome, we ran three logistic regression models, each controlling for that SDG indicator at baseline [38]. The first model included all potential covariates alongside the two social protection factors. The second model retained covariates and social protection factors significant at p < .10, and the third model included only those covariates and social protection factors significant at p < .05. Fourth, we checked for potential effect modifications, arising from interactions between cash and care social protections, and between them and covariates. Using the third, final model for each SDG indicator (, we ran multivariable regressions with interaction terms for cash and care, for cash and any significant covariate, and for care and any significant covariate. Fifth, significant instances of social protection predictors and covariates were entered into marginal effects analyses in STATA. This demonstrated how the predicted probability of the outcome may change when cash or care were accessed, as well as showing potential additive effects of cash plus care, whilst holding covariates at mean values. For SDG 5.6 (pregnancy or childbirth), results are shown only for girls.

## Results

### Adolescents deceased or lost to follow-up

Adolescents deceased or lost to follow-up (n = 114, 3.3%) did not differ at baseline from those retained on rates of TB, school dropout, sexual violence/exploitation, pregnancy or violence perpetration, receipt of ‘cash’ or ‘care’. However, they were more food insecure (χ^2^ = 8.99, *p* = 0.003) and reported more HIV-risks (χ^2^ = 7.26, *p* = 0.007), substance use (χ^2^ = 8.18, *p* = 0.004), and mental health disorder (χ^2^ = 5.15, *p* = 0.023). Although a one-year follow-up of 96.8% is extremely high, some of the most vulnerable participants were deceased or untraceable and thus findings may slightly under-estimate risks.

### Socio-demographics, SDG indicators and social protection

**As shown in** Table 1, the sample was 56.7% female and food insecurity was 34.7% (girls) and 29.1% (boys). Rates of past-year HIV-risk behaviour and mental health disorder ranged from 11.0–20.5%. School dropout was 4.9% (girls) and 3.5% (boys) and past-year sexual violence was 10.1% (girls) and 5.9% (boys). Self-reported violence perpetration was 9.3% (girls) and 13.9% (boys). Receipt of social protection was equal for girls and boys: ‘cash’ alone 43.0/43.3%, ‘care’ alone 10.3/10.8%, and ‘cash plus care’ 34.9/31.5%.

**Table 1:** Socio-demographic characteristics of the sample.

	**Girls (n = 1926)**	**Boys (n = 1475)**
**Sustainable Development Goals**		
2.1 Hunger (1 or more day/week)	668 (34.7%)	429 (29.1%)***
3.3 HIV risk behaviour	262 (13.6%)	302 (20.5%)***
3.4 Tuberculosis	60 (3.1%)	31 (2.1%)
3.4 Mental health risk	281 (14.6%)	162 (11.0%)**
3.5 Substance Abuse	106 (5.5%)	143 (9.7%)***
4.1 School non-enrolment	95 (4.9%)	52 (3.5%)*
5.2 Sexual violence or exploitation	195 (10.1%)	87 (5.9%)***
5.6 Pregnancy	71 (3.7%)	-
16.1 Violence perpetration	180 (9.3%)	205 (13.9%)***
**Social protection**		
Cash only	829 (43.0%)	639 (43.3%)
Care only	199 (10.3%)	159 (10.8%)
Cash plus care	672 (34.9%)	464 (31.5%)*
**Covariates**		
Adolescent aged over 13	936 (48.6%)	705 (47.8%)
Rural location	969 (50.3%)	712 (48.3%)
3 or more children in the home	948 (49.2%)	650 (44.1%)**
2 or more basic necessities missing	1137 (59.0%)	845 (57.3%)
Informal housing	624 (32.4%)	444 (30.1%)
Job in household	1454 (75.5%)	1136 (71.1%)
Child has birth certificate	1829 (95.0%)	1414 (95.9%)
Female primary caregiver	1759 (91.3%)	1297 (87.9%)**
	**Girls (n = 1926)**	**Boys (n = 1475)**
**Sustainable Development Goals**		
2.1 Hunger (1 or more day/week)	668 (34.7%)	429 (29.1%)***
3.3 HIV risk behaviour	262 (13.6%)	302 (20.5%)***
3.4 Tuberculosis	60 (3.1%)	31 (2.1%)
3.4 Mental health risk	281 (14.6%)	162 (11.0%)**
3.5 Substance Abuse	106 (5.5%)	143 (9.7%)***
4.1 School non-enrolment	95 (4.9%)	52 (3.5%)*
5.2 Sexual violence or exploitation	195 (10.1%)	87 (5.9%)***
5.6 Pregnancy	71 (3.7%)	-
16.1 Violence perpetration	180 (9.3%)	205 (13.9%)***
**Social protection**		
Cash only	829 (43.0%)	639 (43.3%)
Care only	199 (10.3%)	159 (10.8%)
Cash plus care	672 (34.9%)	464 (31.5%)*
**Covariates**		
Adolescent aged over 13	936 (48.6%)	705 (47.8%)
Rural location	969 (50.3%)	712 (48.3%)
3 or more children in the home	948 (49.2%)	650 (44.1%)**
2 or more basic necessities missing	1137 (59.0%)	845 (57.3%)
Informal housing	624 (32.4%)	444 (30.1%)
Job in household	1454 (75.5%)	1136 (71.1%)
Child has birth certificate	1829 (95.0%)	1414 (95.9%)
Female primary caregiver	1759 (91.3%)	1297 (87.9%)**
	Girls (n = 1926)	Boys (n = 1475)
Sustainable Development Goals		
2.1 Hunger (1 or more day/week)	668 (34.7%)	429 (29.1%)***
3.3 HIV risk behaviour	262 (13.6%)	302 (20.5%)***
3.4 Tuberculosis	60 (3.1%)	31 (2.1%)
3.4 Mental health risk	281 (14.6%)	162 (11.0%)**
3.5 Substance Abuse	106 (5.5%)	143 (9.7%)***
4.1 School non-enrolment	95 (4.9%)	52 (3.5%)*
5.2 Sexual violence or exploitation	195 (10.1%)	87 (5.9%)***
5.6 Pregnancy	71 (3.7%)	-
16.1 Violence perpetration	180 (9.3%)	205 (13.9%)***
Social protection		
Cash only	829 (43.0%)	639 (43.3%)
Care only	199 (10.3%)	159 (10.8%)
Cash plus care	672 (34.9%)	464 (31.5%)*
Covariates		
Adolescent aged over 13	936 (48.6%)	705 (47.8%)
Rural location	969 (50.3%)	712 (48.3%)
3 or more children in the home	948 (49.2%)	650 (44.1%)**
2 or more basic necessities missing	1137 (59.0%)	845 (57.3%)
Informal housing	624 (32.4%)	444 (30.1%)
Job in household	1454 (75.5%)	1136 (71.1%)
Child has birth certificate	1829 (95.0%)	1414 (95.9%)
Female primary caregiver	1759 (91.3%)	1297 (87.9%)**

*** *p* < .001

** *p* < .01

* *p* < .05.

*p* values associated with Chi Square tests.

### Associations of social protection and SDG health indicators

Logistic regression models, disaggregated by gender, showed that social protection was associated with reduced risks in 12 of 17 measured health-related indicators pertaining to five SDG goals. For pulmonary tuberculosis, no associations were shown for either boys or girls. Regressions controlled for covariates and baseline SDG risk. For five SDG indicators (HIV-risk behaviour, tuberculosis, substance use, school non-enrolment and hunger) patterns of association with social protection showed similar risk-reduction effects amongst boys and girls. For sexual violence, risk-reduction associations were only shown for girls, and for violence perpetration and mental health, risk-reduction associations were only shown for boys. There were no associations showing social protection increasing risk for either gender.

For boys (see Table 2), cash social protection was significantly associated with five SDG indicators: reduced HIV-risk behaviour (OR 0.69 CI 0.50–0.95); reduced mental health disorder (OR 0.67 CI 0.47–0.96); reduced substance use (OR 0.61 CI 0.42–0.89); reduced school dropout (OR 0.10 CI 0.05–0.19) and reduced violence perpetration (OR 0.67 CI 0.48–0.93). Care social protection was significantly associated with four SDG indicators: reduced hunger (OR 0.50 CI 0.34–0.73); reduced HIV-risk behaviour (OR 0.56 CI 0.41–0.77); reduced substance use (OR 0.36 CI 0.23–0.57) and reduced violence perpetration (OR 0.59 CI 0.43–0.81) (Table 2).

**Table 2:** Logistic regression models showing associations between social protection receipt and SDG indicators among boys (N = 1475).

**Step 1**	**SDG 2.1 Hunger**		**SDG 3.3 AIDS**		**SDG 3.3 TB**		**SDG 3.4 Mental Health**		**SDG 3.5 Substance Abuse**		**4.1 School non- enrolment**		**5.2 Sexual violence or exploitation**		**16.1 Violence perpetration**	
	AOR	p	AOR	p	AOR	p	AOR	p	AOR	p	AOR	p	AOR	p	AOR	p
	(95% CI)		(95% CI)		(95% CI)		(95% CI)		(95% CI)		(95% CI)		(95% CI)		(95% CI)	
Cash	1.03	.841	**0.67**	**.017**	0.78	.56	**0.71**	**0.074**	**0.57**	**.006**	**0.11**	**< .001**	0.85	.535	**0.67**	**.019**
	(0.78–1.36)		**(0.48–0.93)**		(0.34–1.81)		**(0.48–1.04)**		**(0.39–0.85)**		**(0.10–0.21)**		(0.52–1.41)		**(0.47–0.94)**	
Care	**0.74**	**.011**	**0.58**	**.001**	0.66	.661	0.76	0.138	**0.37**	**< .001**	**0.48**	**.063**	0.67	.107	**0.61**	**.004**
	**(0.58–0.93)**		**(0.42–0.79)**		(0.31–1.43)		(0.53–1.09)		**(0.23–0.57)**		**(0.23–1.04)**		(0.42–1.09)		**(0.44–0.86)**	
Baseline outcome	**1.98**	**< .001**	**5.5**	**< .001**	**6.38**	**< .001**	**2.59**	**< .001**	**2.12**	**< .001**	**50.18**	**< .001**	**3.28**	**.002**	**1.59**	**.009**
	**(1.56–2.51)**		**(3.86–7.85)**		**(2.56–15.90)**		**(1.73–3.90)**		**(1.45–2.09)**		**(13.77–182.94)**		**(1.54–6.96)**		**(1.13–2.24)**	
Older than 13 years	1.17	.172	**4.19**	**< .001**	0.69	.340	1.22	0.258	**3.67**	**< .001**	1.57	.192	**2.63**	**< .001**	**1.44**	**.021**
	(0.93–1.48)		**(3.03–5.80)**		(0.32–1.48)		(0.86–1.72)		**(2.41–5.59)**		(0.80–3.10)		**(1.60–4.32)**		**(1.06–1.97)**	
Urban location	**1.6**	**< .001**	1.13	.414	0.73	.731	0.81	0.252	1.29	.190	1.35	.368	0.96	.867	1.09	.579
	**(1.27–2.02)**		(0.84–1.53)		(0.34–1.58)		(0.57–1.15)		(0.88–1.89)		(0.70–2.58)		(0.60–1.53)		(0.80–1.50)	
>3 children in home	0.98	.856	0.78	.100	2.05	.060	0.94	0.735	**0.64**	**.027**	**1.12**	**.746**	0.87	.547	0.82	.202
	(0.78–1.23)		(0.58–1.05)		(0.97–4.33)		(0.67–1.33)		**(0.43–0.95)**		**(0.57–2.17)**		(0.55–1.37)		(0.60–1.12)	
Has birth certificate	0.85	.562	1.07	.853	0.33	.094	0.83	0.64	0.68	.346	0.69	.527	1.28	.696	**0.51**	**.035**
	(0.49–1.48)		(0.52–2.21)		(0.09–1.21)		(0.39–1.80)		(0.31–1.52)		(0.22–2.19)		(0.37–4.37)		**(0.27–0.95)**	
Female caregiver	0.87	.411	0.94	.787	1.16	.819	0.86	0.541	1.33	.309	0.59	.192	0.68	.203	1.18	.488
	(0.62–1.22)		(0.62–1.43)		(0.34–3.99)		(0.54–1.39)		(0.77–2.33)		(0.26–1.31)		(0.38–1.23)		(0.74–1.88)	
Missing >2 necessities	**1.96**	**< .001**	1.12	.473	1.87	.140	1.3	0.173	**0.95**	**.810**	**3.53**	**.002**	0.78	.295	0.99	.942
	**(1.52–2.53)**		(0.82–1.54)		(0.82–4.28)		(0.89–1.88)		**(0.64–1.41)**		**(1.58–7.86)**		(0.49–1.25)		(0.71–1.37)	
Informal housing	**1.58**	**< .001**	**1.43**	**.026**	0.67	.384	1.4	0.07	1.18	.414	1.76	.092	0.69	.176	1.18	.342
	**(1.23–2.03)**		**(1.05–1.97)**		(0.27–1.66)		(0.97–2.01)		(0.79–1.75)		(0.91–3.41)		(0.41–1.18)		(0.84–1.64)	
Job in household	0.83	.841	0.94	.528	1.44	.104	0.98	0.882	1.01	.912	0.74	.162	1.11	.440	1.15	.139
	(0.72–0.95)		(0.78–1.13)		(0.93–2.23)		(0.80–1.22)		(0.80–1.28)		(0.49–1.13)		(0.85–1.47)		(0.96–1.39)	
Constant	**0.23**	**< .001**	**0.1**	**< .001**	**0.04**	**0.004**	**0.2**	**0.004**	**0.06**	**< .001**	**0.06**	**0.003**	**0.06**	**< .001**	**0.25**	**0.005**
**Step 2**	**SDG 2.1 Hunger**		**SDG 3.3 AIDS**		**SDG 3.3 TB**		**SDG 3.4 Mental Health**		**SDG 3.5 Substance Abuse**		**4.1 School non- enrolment**		**5.2 Sexual violence or exploitation**		**16.1 Violence perpetration**	
	AOR	p	AOR	p	AOR	p	AOR	p	AOR	p	AOR	p	AOR	p	AOR	p
	(95% CI)		(95% CI)		(95% CI)		(95% CI)		(95% CI)		(95% CI)		(95% CI)		(95% CI)	
Cash	-	-	**0.71**	**.033**	-	-	**0.67**	**.027**	**0.61**	**0.011**	**0.1**	**< .001**	-	-	**0.67**	**.016**
			**(.52-.97)**				**(0.47–0.96)**		**(0.42–0.89)**		**(0.05–0.19)**				**(0.48–0.93)**	
Care	**0.73**	**.008**	**0.57**	**< .001**	-	-	-	-	**0.36**	**< .001**	0.5	.072	-	-	**0.59**	**.001**
	**(0.58–0.92)**		**(.41-.77)**						**(0.23–0.57)**		(0.24–1.06)				**(0.43–0.81)**	
Baseline outcome	**1.98**	**< .001**	**5.46**	**< .001**	**-**	**-**	**2.78**	**< .001**	**2.22**	**< .001**	**53.1**	**< .001**	-	-	**1.58**	**.009**
	**(1.57–2.51)**		**(3.83–7.77)**				**(1.87–4.15)**		**(1.53–3.22)**		**(15.12–186.51)**				**(1.12–2.22)**	
Older than 13 years	-	-	**4.23**	**< .001**	-	-	-	-	**3.63**	**< .001**	-	-	-	-	**1.45**	**.017**
			**(3.07–5.84)**						**(2.40–5.51)**						**(1.07–1.98)**	
Urban location	**1.63**	**< .001**	**-**	**-**	-	**-**	-	**-**	-	-	**-**	**-**	-	**-**	-	**-**
	**(1.30–2.05)**															
>3 children in home	**-**	**-**	0.77	.088	**-**	**-**	**-**	**-**	**0.63**	**0.017**	**-**	**-**	**-**	**-**	**-**	**-**
			(.57–1.04)						**(0.42–0.92)**							
Has birth certificate	-	-	-	-	-	-	-	-	**-**	**-**	-	-	-	-	**0.49**	**.025**
															**(0.26–0.91)**	
Female caregiver	-	-	-	-	-	-	-	-	-	-	-	-	-	-	-	-
Missing >2 necessities	**1.95**	**< .001**	-	-	-	-	-	-	-	-	**4.13**	**< .001**	-	-	-	-
	**(1.52–2.51)**										**(1.89–9.00)**					
Informal housing	**1.61**	**< .001**	**1.45**	**.018**	-	-	**1.62**	**.006**	-	-	1.76	.082	-	-	-	-
	**(1.26–2.06)**		**(1.06–1.97)**				**(1.15–2.28)**				(0.93–3.34)					
Job in household	**0.83**	**.010**	-	-	-	-	-	-	-	-	-	-	-	-	-	-
	**(0.72–0.96)**															
Constant	**0.18**	**< .001**	**0.12**	**< .001**	-	-	**0.12**	**< .001**	**0.09**	**< .001**	**0.04**	**< .001**	-	-	**0.38**	**.005**
**Step 3**	**SDG 2.1 Hunger**		**SDG 3.3 AIDS**		**SDG 3.3 TB**		**SDG 3.4 Mental Health**		**SDG 3.5 Substance Abuse**		**4.1 School non- enrolment**		**5.2 Sexual violence or exploitation**		**16.1 Violence perpetration**	
	AOR	p	AOR	p	AOR	p	AOR	p	AOR	p	AOR	p	AOR	p	AOR	p
	(95% CI)		(95% CI)		(95% CI)		(95% CI)		(95% CI)		(95% CI)		(95% CI)		(95% CI)	
Cash	-	-	**0.69**	**.021**	-	-	**0.67**	**.027**	**0.61**	**.011**	**0.1**	**< .001**	-	-	**0.67**	**.016**
			**(0.50–0.95)**				**(0.47–0.96)**		**(0.42–0.89)**		**(0.05–0.19)**				**(0.48–0.93)**	
Care	**0.5**	**< .001**	**0.56**	**< .001**	-	-	-	-	**0.36**	**< .001**	**-**	**-**	-	-	**0.59**	**.001**
	**(0.34–0.73)**		**(0.41–0.77)**						**(0.23–0.57)**						**(0.43–0.81)**	
Baseline outcome	**1.98**	**< .001**	**5.59**	**< .001**	-	-	**2.78**	**< .001**	**2.22**	**< .001**	**56.26**	**< .001**	-	-	**1.58**	**.009**
	**(1.57–2.51)**		**(3.93–7.95)**				**(1.87–4.15)**		**(1.53–3.22)**		**(16.12–196.36)**				**(1.12–2.22)**	
Older than 13 years	-	-	**4.2**	**< .001**	-	-	-	-	**3.63**	**< .001**	**-**	**-**	-	-	**1.45**	**.017**
			**(3.05–5.80)**						**(2.40–5.51)**						**(1.07–1.98)**	
Urban location	**1.59**	**< .001**	-	-	-	-	-	**-**	-	-	**-**	**-**	-	**-**	-	**-**
	**(1.27–2.01)**															
>3 children in home	**-**	**-**	**-**	**-**	**-**	**-**	**-**	**-**	**0.63**	**.017**	**-**	**-**	**-**	**-**	**-**	**-**
									**(0.42–0.92)**							
Has birth certificate	-	-	-	-	-	-	-	-	**-**	**-**	-	-	-	-	**0.49**	**.025**
															**(0.26–0.91)**	
Female caregiver	-	-	-	-	-	-	-	-	-	-	-	-	-	-	-	-
Missing >2 necessities	**1.53**	**.008**	-	-	-	-	-	-	-	-	**5.13**	**< .001**	-	-	-	-
	**(1.12–2.09)**										**(2.40–10.95)**					
Informal housing	**1.61**	**< .001**	**1.5**	**.010**	-	-	**1.62**	**.006**	-	-	-	-	-	-	-	-
	**(1.26–2.05)**		**(1.10–2.03)**				**(1.15–2.28)**									
Job in household	**0.83**	**.010**	-	-	-	-	-	-	-	-	-	-	-	-	-	-
	**(0.72–0.96)**															
Care* Missing >2 necessities	**1.84**	**.013**	**-**	**-**	-	-	-	-	-	-	-	-	-	-	-	-
	**(1.14–2.98)**															
Constant	**0.22**	**< .001**	**0.07**	**< .001**	-	-	**0.12**	**< .001**	**0.09**	**< .001**	**0.03**	**< .001**	-	-	**0.38**	**.005**

*Note*. For each outcome, interactions between (a) cash and care and (b) each of cash and care and the covariates in the model were tested. None were statistically significant except for the interaction between care and missing necessities for the outcome hunger, as shown in step 3, and thus only significant effects are illustrated.

**Fig 1:**
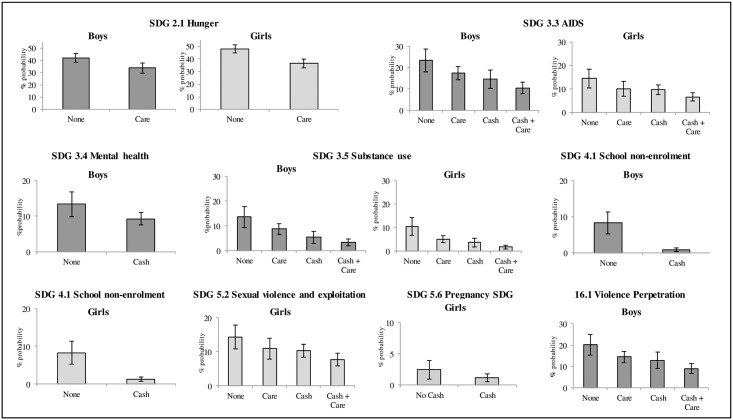
Predicted percent probabilities of SDG indicators when ‘cash,’ ‘care,’ ‘cash and care,’ or ‘no provision’ (‘none’) are received, holding all other covariates in the logistic regression model at their average levels (see Tables 2 and 3 for variables included in final predictive model for each outcome). Error bars indicate 95% confidence intervals.

No interactive effects between cash and care were shown for boys, but additive effects of cash and care were associated with greater risk reductions in three SDG indicators (see Fig 1). Among boys, substance use incidence in the past year was 13.6% without cash and care provision, 8.7% with cash provision, 5.4% with care, and 3.3% with cash plus care. For violence perpetration, incidence was 20.1% without cash and care provision, 14.4% with cash provision, 12.9% with care alone, and 9.0% with cash plus care,. For HIV-risk behaviour, incidence was 23% without cash and care, 17.5% with cash alone, 14.7% with care alone, and 10.6% with cash plus care.

**Fig 2:**
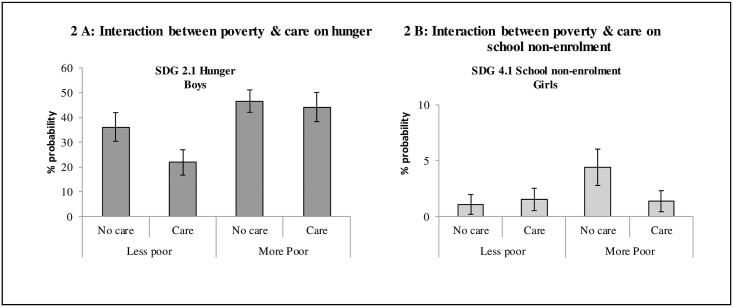
Panel A shows the predicted percent probabilities of adolescent hunger among boys for the interaction between missing necessities and receipt of ‘care’ provisions. Panel B shows the predicted percent probabilities of school non-enrolment among girls for the interaction between missing necessities and receipt of care provisions.

There was only one statistically significant interaction of cash or care with a socio-demographic covariate, among boys: poverty and care on the SDG indicator of hunger. Among boys who were less poor, care had a markedly greater effect on reducing hunger (36.1% reduced to 21.8%) than among boys who were poorer (46.6% reduced to 44.2%) (see Fig 2A).

As shown in Table 3, amongst girls, cash social protection was significantly associated with five SDG indicators: reduced HIV-risk behaviour (OR 0.64 CI 0.46–0.87), reduced substance abuse (OR 0.46 CI 0.30–0.70), reduced school dropout (OR 0.14 CI 0.09–0.24), reduced sexual exploitation (OR 0.67 CI 0.48–0.93), and reduced pregnancy (OR 0.46 CI .27–0.78). Care social protection was significantly associated with four SDG indicators: reduced hunger (p < .001 OR 0.63 CI 0.51–0.77); reduced HIV-risk behaviour (OR 0.67 CI 0.49–0.91); reduced substance use (OR 0.32 CI 0.19–0.53); and reduced sexual exploitation (OR 0.71 CI 0.52–0.98).

**Table 3:** Logistic regression models showing associations between social protection receipt and SDG indicators among girls (N = 1926).

**Step 1**	**SDG 2.1 Hunger**		**SDG 3.3 AIDS**		**SDG 3.3 TB**		**SDG 3.4 Mental Health**		**SDG 3.5 Substance Abuse**		**4.1 School non- enrolment**		**5.2 Sexual violence or exploitation**		**5.6 Pregnancy**		**16.1 Violence perpetration**	
	AOR		AOR		AOR		AOR		AOR		AOR		AOR		AOR		AOR	
	(95% CI)	p	(95% CI)	p	(95% CI)	p	(95% CI)	p	(95% CI)	p	(95% CI)	p	(95% CI)	p	(95% CI)	p	(95% CI)	p
**Cash**	0.98	.887	**0.64**	**.008**	1.09	.809	1.05	.775	**0.45**	**< .001**	**0.13**	**< .001**	**0.68**	**.030**	**0.49**	**.011**	1.13	.554
	(0.77–1.26)		**(0.45–0.89)**		(0.56–2.10)		(0.76–1.45)		**(0.29–0.70)**		**(0.08–0.23)**		**(0.47–0.96)**		**(0.28–0.85)**		(.76–1.68)	
**Care**	**0.63**	**< .001**	**0.69**	**.023**	1.10	.718	0.94	.664	**0.33**	**< .001**	**0.52**	**.017**	**0.71**	**.042**	**0.63**	**.116**	0.92	.922
	**(0.51–0.76)**		**(0.50–0.95)**		(0.65–1.88)		(0.71–1.24)		**(0.20–0.55)**		**(0.30–0.89)**		**(0.51–0.99)**		**(0.36–1.12)**		(.67–1.28)	
**Baseline outcome**	**2.06**	**< .001**	**7.23**	**< .001**	1.94	.115	**3.31**	**< .001**	**2.16**	**< .001**	**7.46**	**< .001**	**4.07**	**< .001**	**15.77**	**< .001**	1.31	.235
	(1.69–2.53)		**(5.06–10.34)**		(0.85–4.43)		**(2.49–4.40)**		**(1.40–3.34)**		**(3.30–16.88)**		**(2.74–6.06)**		**(7.75–32.09)**		(.84–2.06)	
**Older than 13 years**	1.10	.352	**4.45**	**< .001**	0.85	.560	**1.27**	**.089**	**2.33**	**< .001**	**2.74**	**.001**	**2.49**	**< .001**	**11.58**	**< .001**	1.24	.187
	(0.90–1.34)		**(3.07–6.45)**		(0.50–1.45)		**(0.97–1.66)**		**(1.47–3.70)**		**(1.54–4.86)**		**(1.76–3.53)**		**(4.12–32.58**		(.91–1.70)	
**Urban location**	**1.38**	**.002**	1.12	.485	0.74	.276	0.82	.154	0.88	.547	1.20	.462	1.14	.434	0.72	.258	1.20	.267
	**(1.13–1.69)**		(0.82–1.52)		(0.43–1.27)		(0.63–1.08)		(0.58–1.34)		(0.73–1.97)		(0.82–1.58)		(0.41–1.27)		(.87–1.66)	
**>3 children in home**	1.08	.433	**0.77**	**.092**	**1.78**	**.038**	0.87	.302	**0.66**	**.056**	1.01	.975	0.99	.972	0.72	.239	**0.61**	**.002**
	(0.89–1.32)		**(0.57–1.04)**		**(1.03–3.05)**		(0.67–1.13)		**(0.43–1.01)**		(0.62–1.65)		(0.73–1.36)		(0.42–1.24)		**(.44-.83)**	
**Has birth certificate**	1.05	.832	1.21	.546	0.69	.489	0.65	.647	1.24	.620	**0.39**	**.008**	0.86	.635	0.83	.677	1.03	.935
	(0.67–1.64)		(0.65–2.24)		(0.24–2.00)		(0.38–1.09)		(0.53–2.86)		**(0.20–0.78)**		(0.46–1.60)		(.34–2.04)		(.50–2.12)	
**Female caregiver**	0.92	.617	0.96	.879	1.94	.115	1.24	.378	1.11	.781	0.81	.596	0.73	.216	2.67	.131	1.46	.229
	(0.65–1.29)		(0.58–1.59)		(0.85–4.43)		(0.77–2.00)		(0.55–2.23)		(0.38–1.75)		(0.45–1.20)		(.75–9.52)		(.79–2.69)	
**Missing >2 necessities**	**1.86**	**< .001**	1.29	.133	1.33	.329	1.22	.185	1.25	.342	**2.22**	**.008**	1.21	.284	1.22	.511	1.01	.948
	**(1.50–2.31)**		(0.93–1.79)		(0.75–2.34)		(0.91–1.63)		(0.79–1.98)		**(1.24–4.00)**		(0.86–1.70)		(0.68–2.21)		(.72–1.42)	
**Informal housing**	**1.77**	**< .001**	0.95	.767	**0.46**	**.028**	1.04	.797	1.04	.854	1.23	.437	0.78	.181	**0.40**	**.007**	1.01	.621
	**(1.43–2.19)**		(0.68–1.33)		**(0.23–0.92)**		(0.78–1.39)		(0.67–1.64)		(0.73–2.07)		(0.55–1.12)		**(0.20–0.78)**		(.77–1.54)	
**Job in household**	0.95	.379	0.93	.475	0.98	.899	0.95	.545	1.02	.872	0.67	.672	0.98	.845	0.78	.174	0.98	.827
	(0.84–1.07)		(0.77–1.13)		(0.72–1.33)		(0.81–1.12)		(0.79–1.33)		(0.48–0.94)		(0.81–1.19)		(0.55–1.11)		(.81–1.17)	
**Constant**	**0.23**	**< .001**	**0.51**	**< .001**	**0.08**	**.004**	**0.18**	**< .001**	**0.06**	**< .001**	**0.15**	**.013**	**0.10**	**< .001**	**0.01**	**< .001**	**0.05**	**< .001**
**Step 2**	**SDG 2.1 Hunger**		**SDG 3.3 AIDS**		**SDG 3.3 TB**		**SDG 3.4 Mental Health**		**SDG 3.5 Substance Abuse**		**4.1 School non- enrolment**		**5.2 Sexual violence or exploitation**		**5.6 Pregnancy**		**16.1 Violence perpetration**	
	AOR		AOR		AOR		AOR		AOR		AOR		AOR		AOR		AOR	
	(95% CI)	p	(95% CI)	p	(95% CI)	p	(95% CI)	p	(95% CI)	p	(95% CI)	p	(95% CI)	p	(95% CI)	p	(95% CI)	p
**Cash**	-	-	**0.66**	**.013**	-	-	-	-	**0.46**	**< .001**	**0.14**	**< .001**	**0.67**	**.018**	**0.46**	**.004**	-	-
			**(0.48–0.92)**						**(0.30–0.70)**		**(0.09–0.23)**		**(0.48–0.93)**		**(0.27–0.78)**			
**Care**	**0.63**	**< .001**	**0.67**	**.012**	-	-	-	-	**0.32**	**< .001**	**0.50**	**.011**	**0.71**	**.038**	**-**	-	-	-
	**(0.51–0.77)**		**(0.49–0.92)**						**(0.19–0.53)**		**(0.29–0.85)**		**(0.52–0.98)**					
**Baseline outcome**	**2.07**	**< .001**	**7.36**	**< .001**	-	-	-	-	**2.20**	**< .001**	**7.84**	**< .001**	**4.29**	**< .001**	**15.87**	**< .001**	-	-
	**(1.70–2.54)**		**(5.18–10.47)**						**(1.45–3.32)**		**(3.53–17.42)**		**(2.90–6.35)**		**(8.04–31.30)**			
**Older than 13 years**	**-**	**-**	**4.50**	**< .001**	-	-	-	-	**2.32**	**< .001**	**2.76**	**< .001**	**2.53**	**< .001**	**12.13**	**< .001**	-	-
			**(3.10–6.51)**						**(1.47–3.68)**		**(1.56–4.89)**		**(1.79–3.58)**		**(4.33–33.96)**			
**Urban location**	**1.37**	**.002**	-	-	-	-	-	-	**-**	**-**	**-**	**-**	**-**	-	**-**	-	-	-
	**(1.13–1.67)**																	
**>3 children in home**	**-**	**-**	0.77	.083	-	-	-	-	**0.65**	**.050**	**-**	**-**	**-**	-	**-**	-	-	-
			(0.57–1.04)						**(0.43–1.00)**									
**Has birth certificate**	**-**	**-**	-	-	-	-	-	-	-	-	**0.38**	**.006**	**-**	-	**-**	-	-	-
											**(0.20–0.76)**							
**Female caregiver**	**-**	**-**	-	-	-	-	-	-	-	-	**-**	**-**	**-**	-	**-**	-	-	-
**Missing >2 necessities**	**1.87**	**< .001**	-	-	-	-	-	-	-	-	**2.35**	**.004**	**-**	-	**-**	-	-	-
	**(1.51–2.31)**										**(1.32–4.18)**							
**Informal housing**	**1.75**	**< .001**	-	-	-	-	-	-	-	-	**-**	**-**	**-**	-	**0.48**	**.025**	-	-
	**(1.42–2.16)**														**(0.25–0.91)**			
**Job in household**	**-**	**-**	-	-	-	-	-	-	-	-	**.68**	**.020**	**-**	-	**-**	-	-	-
											**(0.49–0.94)**							
**Constant**	**0.23**	**< .001**	**0.07**	**< .001**	-	-	-	-	**0.08**	**< .001**	**.17**	**.001**	**0.08**	**< .001**	**0.01**	**< .001**		
**Step 3**	**SDG 2.1 Hunger**		**SDG 3.3 AIDS**		**SDG 3.3 TB**		**SDG 3.4 Mental Health**		**SDG 3.5 Substance Abuse**		**4.1 School non- enrolment**		**5.2 Sexual violence or exploitation**		**5.6 Pregnancy**		**16.1 Violence perpetration**	
	AOR		AOR		AOR		AOR		AOR		AOR		AOR		AOR		AOR	
	(95% CI)	p	(95% CI)	p	(95% CI)	p	(95% CI)	p	(95% CI)	p	(95% CI)	p	(95% CI)	p	(95% CI)	p	(95% CI)	p
**Cash**	-	-	**0.64**	**.005**	-	-	-	-	**0.46**	**< .001**	**0.14**	**< .001**	**0.67**	**.018**	**0.46**	**.004**	-	-
			**(0.46–0.87)**						**(0.30–0.70)**		**(0.09–0.24)**		**(0.48–0.93)**		**(0.27–0.78)**			
**Care**	**0.63**	**< .001**	**0.67**	**.010**	-	-	-	-	**0.32**	**< .001**	1.49	.439	**0.71**	**.038**	-	-	-	-
	**(0.51–0.77)**		**(.49-.91)**						**(0.19–0.53)**		(0.54–4.10)		**(0.52–0.98)**					
**Baseline outcome**	**2.07**	**< .001**	**7.30**	**< .001**	-	-	-	-	**2.20**	**< .001**	**8.60**	**< .001**	**4.29**	**< .001**	**15.87**	**< .001**	-	-
	**(1.70–2.54)**		**(5.14–10.39)**						**(1.45–3.32)**		**(3.80–19.49)**		**(2.90–6.35)**		**(8.04–31.30)**			
**Older than 13 years**	-	-	**4.44**	**< .001**	-	-	-	-	**2.32**	**< .001**	**2.82**	**< .001**	**2.53**	**< .001**	**12.13**	**< .001**	-	-
			**(3.07–6.43)**						**(1.47–3.68)**		**(1.59–5.00)**		**(1.79–3.58)**		**(4.33–33.96)**			
**Urban location**	**1.37**	**.002**	-	-	-	-	-	-	-	-	-	-	-	-	-	-	-	-
	**(1.13–1.67)**																	
**>3 children in home**	-	-	-	-	-	-	-	-	**0.65**	**.050**	-	-	-	-	-	-	-	-
									**(0.43–1.00)**									
**Has birth certificate**	-	-	-	-	-	-	-	-	-	-	**0.35**	**.003**	-	-	-	-	-	-
											**(0.18–0.70)**							
**Female caregiver**	-	-	-	-	-	-	-	-	-	-	-	-	-	-	-	-	-	-
**Missing >2 necessities**	**1.87**	**< .001**	-	-	-	-	-	-	**-**	-	**4.51**	**.001**	-	-	-	-	-	-
	**(1.51–2.31)**										**(1.93–10.56)**							
**Informal housing**	**1.75**	**< .001**	-	-	-	-	-	-	-	-	-	-	-	-	**0.48**	**.025**	-	-
	**(1.42–2.16)**														**(0.25–0.91)**			
**Job in household**	-	-	-	-	-	-	-	-	-	-	**0.69**	**.024**	-	-	-	-	-	-
											**(0.49–0.95)**							
Care*Missing >2 necessities	-	-	-	-	-	-	-	-	-	-	**0.20**	**.012**	-	-	-	-	-	-
											**(0.06–0.70)**							
**Constant**	**0.23**	**< .001**	**0.07**	**< .001**	-	-	-	-	**0.08**	**< .001**	**0.10**	**< .001**	**0.08**	**< .001**	**0.01**	**< .001**	-	-

*Note*. For each outcome, interactions between (a) cash and care and (b) each of cash and care and the covariates in the model were tested. None were statistically significant except for the interaction between care and missing necessities for the outcome hunger, as shown in step 3, and thus only significant effects are illustrated.

No interactive effects between cash and care were shown for girls, but additive effects of cash and care were associated with maximised risk reductions in three SDG indicators (see Fig 1). Among girls, substance use incidence in the past year was 10.5% without cash or care provision, 5.1% with cash provision, 3.6% with care, and 1.7% with cash plus care. For HIV-risk behaviour, incidence was 14.5% without cash and care, 10.1% with cash provision, 9.7% with care, and 6.7% with cash plus care. For sexual exploitation, incidence was 14.3%, without cash or care, 10.9% with cash provision, 10.3% with care and 7.7% with cash plus care.

There was only one statistically significant interaction of cash or care with a socio-demographic covariate: poverty x care on the SDG indicator of school dropout. Among girls who were poorer, care had a greater effect on reducing school dropout (4.4% to 1.4%) than among girls who were less poor (1.1% to 1.5%) (see Fig 2B).

## Discussion

The SDGs represent opportunities for improving the health of our highest-risk populations. But to achieve these goals, interventions are preferable that have effects across multiple outcomes [17]. This study examines the contribution of social protection provision, a core policy target of the SDGs, on indicators across five SDG goals, amongst deprived South African adolescents.

Findings show a remarkable range of associations with improved SDG outcomes. In 12 of 17 gender-disaggregated indicators cash and/or care were associated with significant reductions in adolescent health-related risks. These positive associations spanned all five goals for which measures were available: hunger (SDG 2), health (SDG 3), education (SDG 4), gender equality (SDG 5) and peaceful societies (SDG 16). Thus, social protection seems to positively impact multiple domains of adolescent health and wellbeing.

This study also provides evidence on components and combinations of social protection. Cash support had independent risk reduction effects for ten SDG indicators, and care support had independent risk reduction effects for eight SDG indicators. However, for many SDG health-related targets, strong additive effects were shown of combining cash plus care: with cumulative risk reduction effects shown on six SDG indicators. Overall, combination social protection may be an effective way to maximise health and wellbeing benefits for high-risk adolescents.

There are a number of potential mechanisms for the impacts of social protection on multiple health-related outcomes. Prior research has shown that cash transfers work in multiple ways within a household and are primarily used by families for food and education costs [39]. Qualitative and quantitative evidence suggests that they reduce need for adolescents to have transactional sexual relationships with older partners who provide food and essential financial support to the household [12]. There is also evidence that improved supervision and monitoring of adolescents is associated with lower exposure to community violence, risk of sexual exploitation and pregnancy [40]. Finally, evidence from the parenting support literature has demonstrated that improved caregiver-child relationships can lead to improved mental health outcomes within the family [41]. Evidence regarding combination ‘cash plus care’ mechanisms is still emerging, but one study of HIV risk reduction suggests that the two types of support work in complementary ways and may target different stages in pathways to risk–for example cash reduces the impact of structural deprivations on family psychososial problems (such as abuse, child behaviour problems), and care targets those psychosocial problems directly [42]. Elucidating the mechanisms of cash plus care for other outcomes is clearly an area for future research.

In the course of checking for possible interaction effects, it emerged that the effect of care provision may be modified by adolescents’ level of poverty. Amongst boys, care provision was associated with reduced hunger, but only for those at lower levels of poverty. It may be that improved parental care resulted in greater food allocation to children in the family, but where poverty was very severe, even the most caring of parents did not have opportunities to reduce their adolescents’ hunger levels. Amongst girls, those living in severe poverty were most likely to drop out of school, but for these girls, care provision had the greatest effect, reducing school dropout substantially. It may be that–with largely free schooling in these low-income areas–girls living in high poverty levels require psychosocial support in order to be able to engage with education. We note however that two significant interactions in a large set of possible interactions tested may be due to chance and that future research would be needed to confirm these findings.

Findings also show that social protection is not a panacea. There were no statistically significant associations with indicators of tuberculosis, nor with girls’ mental health or violence perpetration, nor with boys’ victimisation by sexual violence, suggesting that more specified interventions are required. These may require greater expertise and targeting. As patterns of effect differ by gender, social protection may also require a gendered understanding to inform and target combinations of interventions.

There are a number of important limitations to these findings. First, social protection access was not randomized, and although analyses carefully controlled for a range of potential confounders, this observational study does not provide the level of causal reliability of a randomized trial. We attempted to mitigate this risk by checking for, interaction effects among social protections and with significant socio-demographic covariates. Only two such instances were found, in which the impact of social protection differed by poverty level. Further research using fixed effects models may be desirable, to better examine how changes in access to social protection over time are associated with SDG outcomes. Third, the study only has two annual time-points, which again limits the capacity for causal certainty. It will be essential to examine longer-term associations of social protection and SDG outcomes as adolescents progress into adulthood. Fourth, research could valuably examine the levels and length of social protection exposure required in order to reduce SDG risks. Fifth, this study only takes place in one country and initially in two provinces. However, a systematic sampling method was followed in both urban and rural areas, with rigorous follow-up. Further research across other low and middle-income countries is required in order to understand whether a South African experience is indicative of other settings in the region or elsewhere. Sixth, the study only examined indicators in five of the SDG goals, focusing on those that were most directly relevant to adolescent health. Other SDGs, for example SDG 8 ‘economic growth, full and productive employment, and decent work for all’ become increasingly important as adolescents become adults. SDG 6 ‘sustainable water and sanitation for all’ is a key health-related factor, but is provided in South Africa at a large-scale level by government services and was therefore likely to be weakly associated with the household-focused social protection provisions examined in this study. SDGs 1 and 10 ‘end poverty’ and ‘reduce inequality’ are broad and multidimensional concepts that include the aims of other SDGs such as health, education and food security, but are essential overarching considerations in health. Finally, in any testing of multiple possible outcomes, there is a risk that findings are due to chance. To mitigate this, while we have reported findings of .05<p < .01 in the tables for their substantive interest, we note that more than three-quarters of findings for boys and girls alike are significant at the more stringent p< = .01 level. Similarly, the two significant interactions among the large number tested may be due to chance and further research would be needed to confirm this possible effect modification.

Despite these limitations, this study has notable advantages, particularly in external validity. It measured real-world interventions provided by an African government, NGOs, and families. The longitudinal data allowed analyses to examine incident outcomes by controlling for baseline SDG health risks, thus providing stronger causal assumptions. We know that, unlike carefully controlled experimental situations, implementation of any social programmes in Sub-Saharan Africa is administratively challenging and fraught with logistical problems. But, even in these conditions, and amongst a population of young people who are hardest-hit by poverty and inequality, social protection provision was associated with substantial positive impacts on adolescent health and wellbeing.

These findings demonstrate the potential of social protection to contribute to multiple aspects of the SDG agenda. They highlight the value of providing ‘care’ as well as ‘cash’, suggesting the importance of resource allocation to psychosocial care in a time of global cuts and reliance on NGO and soft providers. It is clear that social protection does not solve every societal problem and that for key outcomes such as tuberculosis and mental health we will need additional, targeted investments. But the wide-ranging impacts of social protection also provide a major opportunity. The last decade of programming for children has seen a growth in global initiatives that provide a blueprint on how evidence can be corralled, synthesized, and used as a key driver of policy.[43, 44] There is no question that the Sustainable Development Goals present a challenging set of aspirations. But they also include the potential to contribute to the health of our next generations.
